# Advanced Glycation End Product (AGE)-AGE Receptor (RAGE) System Upregulated Connexin43 Expression in Rat Cardiomyocytes via PKC and Erk MAPK Pathways

**DOI:** 10.3390/ijms14022242

**Published:** 2013-01-24

**Authors:** Lu Yu, Yanbo Zhao, Shengjie Xu, Fang Ding, Chongying Jin, Guosheng Fu, Shaoxiang Weng

**Affiliations:** Department of Cardiovascular Medicine, Sir Run Run Shaw Hospital, College of Medicine, Zhejiang University, Hangzhou 310016, China; E-Mails: jyulu@sina.com.cn (L.Y.); zyboshimly@163.com (Y.Z.); xusj@zju.edu.cn (S.X.); dingdinghello@sina.com (F.D.); jincy_lv@163.com (C.J.); fuguoshengvip@sina.com (G.F.)

**Keywords:** advanced glycation end product, connexin43, cardiomyocytes, diabetes

## Abstract

The remodeling of cardiac gap junction contributes to the arrhythmias in a diabetic heart. We previously reported that high glucose reduced Cx43 protein level in neonatal rat cardiomyocytes. But, the effect and mechanisms of advanced glycation end product (AGE) on Cx43 expression still remain unclear. In this study, we measured the AGE receptor (RAGE) and Cx43 expression by immunohistochemisty in AGE-infused Sprague-Dawley (SD) rats. *In vitro*, the Cx43 and RAGE levels were detected in AGE-treated cardiomyocytes by Western blot and real-time RT-PCR. The function of cells coupling was measured by Scrap loading dye transfer assay. Our results showed that the AGE-infused rat hearts exhibited increased cardiac RAGE and Cx43, as well as Cx43 redistribution. In cultured cardiomyocytes, AGE elevated RAGE expression in a time- and dose-dependent manner. Cx43 protein and mRNA levels were upregulated by AGE (200 mg/L, 24 h), but the gap junction function was not enhanced. RAGE-targeted knock-down or the addition of PKC, and Erk inhibitors abolished the effect of AGE on Cx43. Therefore, AGE-RAGE system might elevate Cx43 expression in rat cardiomyocytes by activating PKC and Erk MAPK pathways, and it also enhanced Cx43 redistribution *in vivo*, which might contribute to the arrhythmias in diabetes.

## 1. Introduction

Diabetes contributes to the high incidence of cardiac arrhythmias, including atrial or ventricular fibrillation and sudden death [1]. Recent studies began to focus its mechanisms and indicated that altered cardiac gap junction expression or function could lead to conduction disturbance and arrhythmias [2,3]. It has been demonstrated that Cx43 is the major connexin in ventricular cardiomyocytes, whereas Cx40 and Cx45 is relatively co-expressed in atrial myocytes and the conduction system [4]. Therefore, the quantity alteration or redistribution of Cx43 could provide an arrhythmogenic substrate for various arrhythmias.

Hyperglycemia is regarded as a major initiator for cardiac complications in diabetes [5]. In our previous study [6], we demonstrated that high glucose could reduce Cx43 expression in neonatal rat cardiomyocytes. But, in some other recent studies, the amount of cardiac Cx43 was up- or down-regulated in streptozotocin (STZ)-induced diabetic rat model [7–9]. Therefore, we could not exclude the possibility that the discordant results might be due to other factors other than high glucose alone, such as advanced glycation end product (AGE). AGE are the products of nonenzymatic glycoxidation of proteins accumulated in diabetes and participate in various cardiovascular diseases, mainly through their receptor (RAGE) [10,11]. Recent studies have reported that the AGE and RAGE-ligand interaction was involved in the pathogenesis of cardiomyocytes injury induced by hopoxia/reoxygenation [12], and it also contributed to calcium handling impairment [13] or the onset of diabetic cardiomyopathy [14]. The results indicated the pathogenic effect of the AGE-RAGE system on cardiomyocytes. But, direct evidence is still absent for Cx43 expression under AGE treatment, which might lead to cardiac Cx43 remodeling in diabetic models.

It was also demonstrated that Cx43 distribution could be an important factor for electrical conduction velocity and vulnerability limits [15], which was associated with cardiac arrhythmias. Disorganized connexins and gap junction could not form junction channels [16], and enhanced Cx43 lateralization may reduce cell excitability [17]. To date, several studies [17,18] have pointed out the Cx43 redistribution in a diabetic heart. But the effect of AGE alone still needs more investigation.

Therefore, in the present study, we aimed to explore the effect and mechanisms of AGE on cardiac Cx43/RAGE expression in rat cardiomyocytes and their possible redistribution *in vivo* models.

## 2. Results and Discussion

### 2.1. Results

#### 2.1.1. The Cardiac RAGE and Cx43 Expressions in AGE-Infused Rats

**Figure 1:**
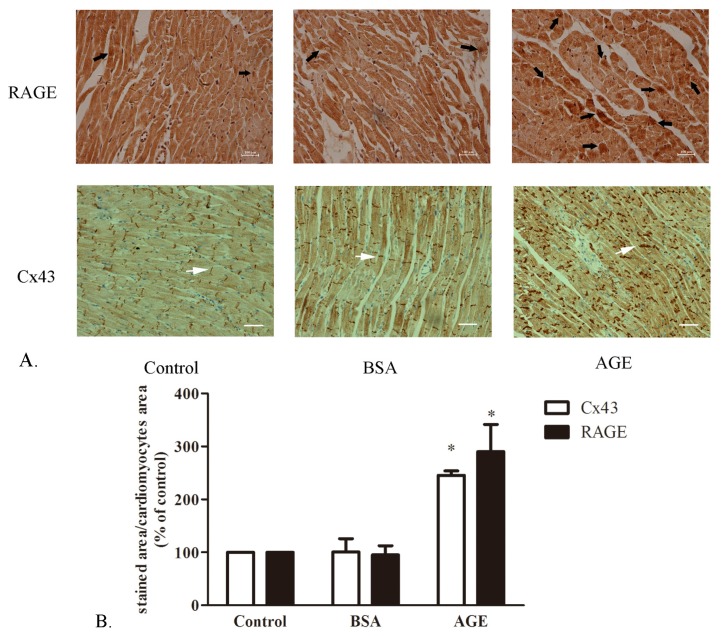
The advanced glycation end product (AGE)-AGE receptor (RAGE) and Cx43 staining in rat heart tissue. (**A**) The RAGE and Cx43 expression detected by immuohistochemisty; (**B**) Quantity analysis of staining assessed by ImageJ software. bovine serum albumin (BSA): BSA-infused rat (40 mg/kg/d); AGE: AGE-infused rat (40 mg/kg/d). Black arrow: RAGE; White arrow: Cx43. Bar: 100 μM. * *p* < 0.05 *vs.* control.

The RAGE and Cx43 staining in BSA- or AGE-infused rat hearts were performed by immunohistochemisty. As displayed in Figure 1, RAGE proteins were expressed abundantly in AGE-infused rat heart, while they were rarely observed in BSA-infused rat or control group. Labeling of cardiac Cx43 also exhibited a significant upregulation and redistribution, which scattered and lateralized along cardiomyocytes. No significant Cx43 staining difference was seen in BSA-infused rat heart compared to control group. The result indicated the upregulation effect of AGE on RAGE and Cx43 protein levels *in vivo* models.

#### 2.1.2. Effects of AGE on Cell Viability

**Figure 2:**
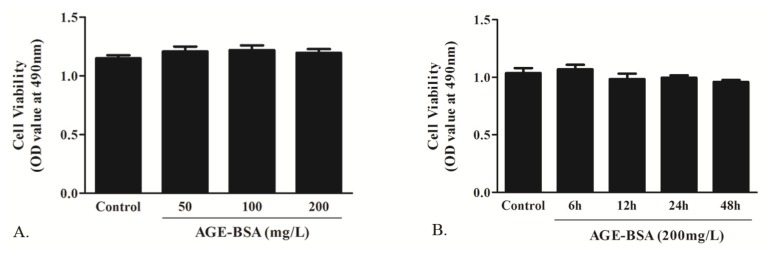
Effect of AGE on the cell viability determined by MTT assay. (**A**) Cells were treated with AGE at the concentration of 50, 100 and 200 mg/L for 24 h; (**B**) Cells were treated with AGE at 200 mg/L for 6, 12, 24 and 48 h respectively. *n* = 5 wells in each individual experiment. * *p* < 0.05 *vs*. control; Data are mean ± SD.

In *in vitro* study, the cultured neonatal rat cardiomyocytes were exposed to AGE treatment. We measured the cell viability under AGE incubation by MTT assay. As a recent study reported [19], cells were treated with 0, 50, 100 and 200 mg/L AGE for 24 h or 0, 6, 12, 24 and 48 h of AGE at a concentration of 200 mg/L. In Figure 2, it showed that AGE had no significant cytotoxic effect on cardiomyocytes.

#### 2.1.3. AGE Upregulated RAGE Expression in Cardiomyocytes

**Figure 3:**
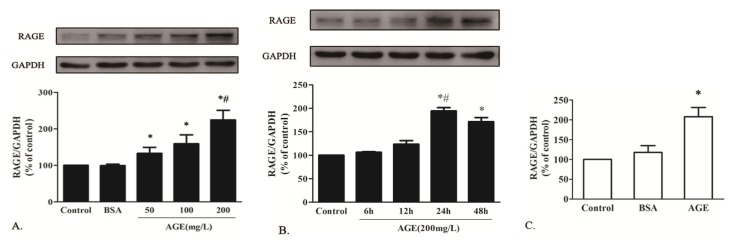
The effect of AGE on RAGE expression in cardiomyocytes. (**A**) AGE upregulated the RAGE protein expression in a dose-dependent manner and peaked at 200 mg/L; (**B**) AGE increased the RAGE protein expression in a time-dependent manner and peaked at 24 h; (**C**) RAGE mRNA level was upregulated by AGE (200 mg/L) treatment for 24 h. * *p* < 0.05 *vs.* control, BSA (200 mg/L); # *p* < 0.05 *vs.* AGE (50 mg/L, 100 mg/L) or AGE (6h, 12h, 48 h); Data are mean ± SD.

To investigate the effect of AGE on RAGE expression *in vitro*, cells were exposed to BSA (200 mg/L) or AGE at various concentrations (50, 100, 200 mg/L) for 24 h. The RAGE protein level was elevated in a dose-dependent manner and peaked at 200 mg/L in cells treated by AGE, whereas BSA alone showed no significant effect on it (Figure 3A). Cells were then treated by AGE at 200 mg/L for 6, 12, 24 and 48 h. RAGE protein also increased in a time-dependent manner and peaked at 24 h (Figure 3B). The real-time RT-PCR also showed that the RAGE mRNA level was elevated by AGE (200 mg/L) treatment for 24 h and remained unchanged in BSA-treated cardiomyocytes (Figure 3C).

#### 2.1.4. The Effects of AGE on Cx43 Expression and GJIC Function in Cardiomyocytes

**Figure 4:**
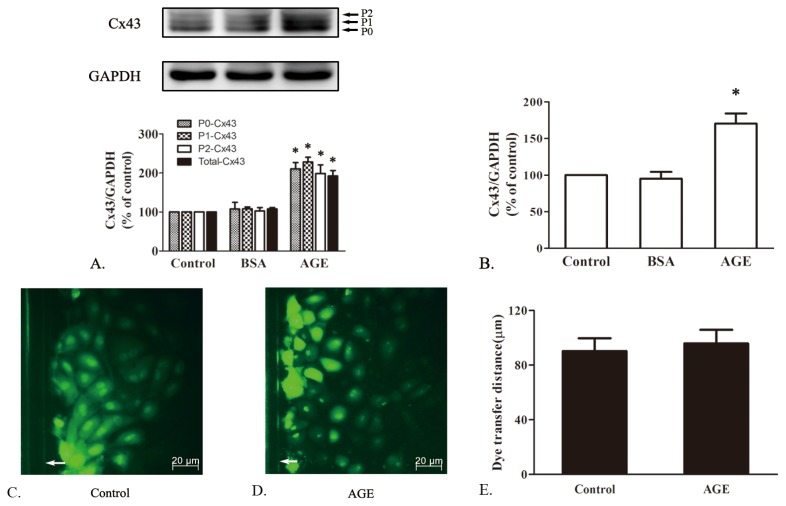
The effect of AGE on Cx43 expression and gap junctional intercellular communication (GJIC) function. (**A**) Cx43 protein (P0, P1, P2) expression was upregulated by AGE (200 mg/L) treatment for 24 h; (**B**) Cx43 mRNA level was upregulated by AGE (200 mg/L) treatment for 24 h. (**C**) and (**D**) showed effect of AGE on the GJIC function assessed by Scrape loading dye transfer assay; (**E**) The quantity analysis of dye transfer distance in each group. * *p* < 0.05 *vs.* control, BSA (200 mg/L); White arrow: scrape line; Data are mean ± SD.

As AGE increased RAGE expression most effectively at 200 mg/L for 24 h, the same concentration and incubation time of AGE or BSA were used in detecting cardiac Cx43. The Cx43 antibody recognizes three bands by Western blots, which consists of a nonphosphorylated form (P0) at 41 kDa and two phosphorylated forms (P1, P2), ranging in size between 43 and 45 kDa. As shown in Figure 4A, AGE upregulated total Cx43 protein expression, including P0, P1 and P2, whereas BSA alone showed no significant effect. The similar change of Cx43 mRNA level could also be observed by real-time RT-PCR (Figure 4B).

Therefore, our results indicated that AGE could elevate RAGE/Cx43 expression both *in vivo* and *in vitro*. Unexpectedly, however, compared to the control group, cells treated by AGE (200 mg/L, 24 h) showed no significant change in the distance of the dye transferred from the scrape margin (Figure 4C–E). It suggested that the AGE-elevated Cx43 might not exert their activity or enhance cellular coupling in cardiomyocytes.

#### 2.1.5. AGE Increased Cx43 Expression via AGE-RAGE Pathway

**Figure 5:**
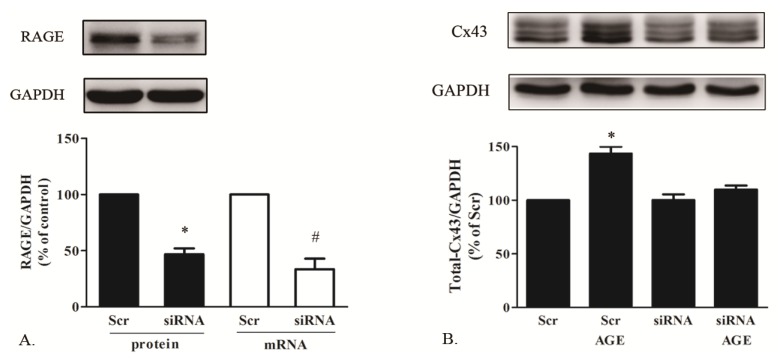
The effect of AGE on Cx43 expression in cardiomyocytes with RAGE knocked down. (**A**) The identification of RAGE expression knocked down with small interfering (siRNA) assessed by Western blot and real-time polymerase chain reaction (PCR); (**B**) The effect of AGE on Cx43 protein in cardiomyocytes with or without RAGE knocked down. * *p* < 0.05 *vs.* Scr (Scrambled siRNA); siRNA: RAGE-targeted siRNA. Data are mean ± SD.

According to the *in vivo* and *in vitro* results, AGE significantly increased cardiac Cx43 expression. But, the mechanisms were still unclear. As RAGE has been indicated as an interaction ligand of AGE in exerting various pathogenic effects, we investigated whether RAGE was involved in this effect. By using siRNA technology, we knocked down the RAGE expression, which was identified by Western blot and real-time RT-PCR (Figure 5A). As seen in Figure 5B, Cx43 was not elevated by AGE incubation in cells with RAGE knocked down, whereas it could still be upregulated in cardiomyocytes with scrambled siRNA treatment. The result suggested that AGE elevated Cx43 level in cardiomyocytes mainly by RAGE activation.

#### 2.1.6. AGE Activated MAPK Pathway

**Figure 6:**
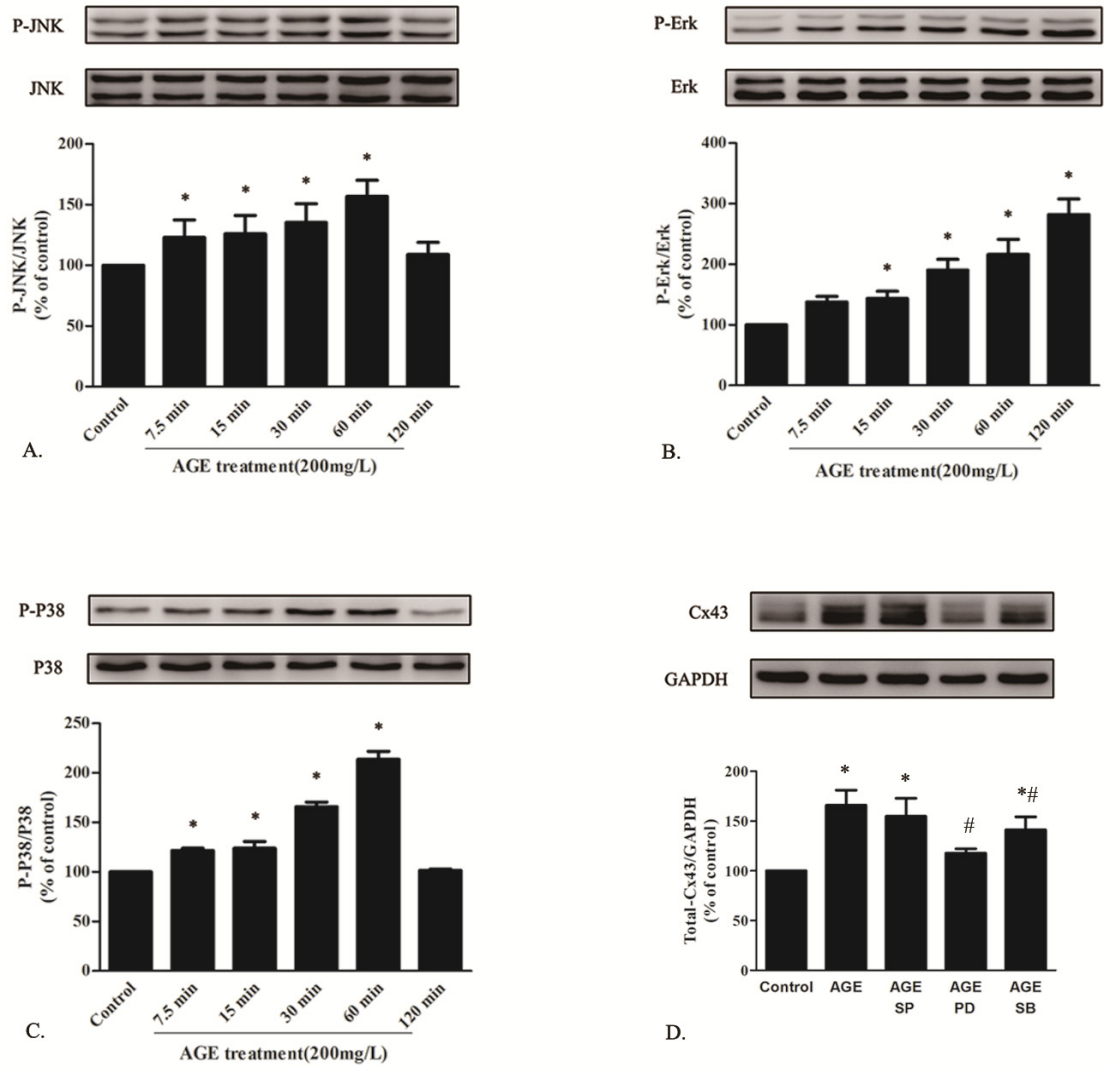
AGE elevated time-dependent phosphorylation levels of JNK (**A**); Erk (**B**) and P38 MAPK (**C**) in cardiomyocytes determined by Western blot. (**D**) Western blot analysis of Cx43 expression in AGE-treated cardiomyocytes with the presence of MAPK inhibitors. * *p* < 0.05 *vs*. control; # *p* < 0.05 *vs.* AGE; Data are mean ± SD.

Activation of RAGE induced by AGE engages various signal transduction pathways, among which MAPKs act as a major one in response to extracellular signals [20]. To investigate AGE-induced signaling pathways, the phosphorylation levels of MAPK family proteins (Erk, p38, JNK) were measured in cardiomyocytes treated by AGE at 200 mg/L for 7.5, 15, 30, 60 and 120 min. As seen in Figure 6A–C, AGE activated the time-dependent phosphorylation level of JNK, Erk and p38 assessed by Western blot.

Cells were then co-incubated by AGE (200 mg/L) or AGE with the presence of JNK MAPK inhibitor SP600125 (20 μM), Erk MAPK inhibitor PD98059 (20 μM) or p38 MAPK inhibitor SB203580 (10 μM) for 24 h. All the inhibitors were added to cells 30 min before AGE treatment. Our previous study had demonstrated that MAPK inhibitors alone did not affect cardiac Cx43. In this present work, we observed that only PD98059 totally abolished the AGE-induced Cx43 upregulation (Figure 6D). Although SB203580 partly downregulated the total Cx43 level increased by AGE, it only alleviated the upregulation of Cx43 in P2 band, whereas it had no effect on the increased expression of P0- or P1-Cx43 (statistical analysis data not shown). Therefore, it could be supposed that AGE elevated Cx43 expression partly by Erk MAPK pathway.

#### 2.1.7. AGE Activated PKC Pathway

**Figure 7:**
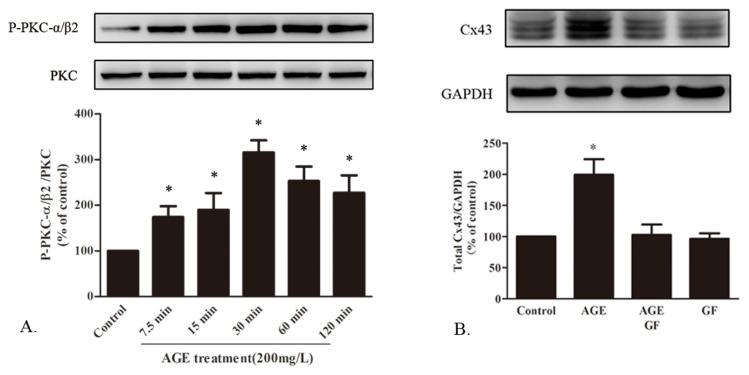
The involvement of the PKC pathway in the effect of AGE on Cx43 expression. (**A**) AGE activated the time-dependent phosphorylation of PKCα/β2 in cardiomyocytes assessed by Western blot; (**B**) Cx43 protein expression in cells treated by AGE for 24 h with the presence of PKC inhibitor GF109203X (GF, 10 μM). * *p* < 0.05 *vs.* control; Data are mean ± SD.

In the progression of diabetes, the PKC pathway is considered as an important signal transduction pathway leading to structural or functional changes in cardiomyocytes. PKC-α and PKC-β2 were reported as the predominant PKC isoforms expressed in the heart tissue [21]. As an upstream signal, the phosphorylation level of PKCα/β2 was detected in cardiomyocytes incubated by AGE for 7.5, 15, 30, 60 and 120 min. Western blot showed that it could be activated by AGE in a time-dependent manner (Figure 7A). The cells were then co-treated by AGE with the presence of PKC inhibitor GF109203X (10 μM), which was added to cells 30 min before the AGE treatment. We noted that GF109203X could totally abrogate the Cx43 upregulation induced by AGE, while GF109203X alone had no significant effect on Cx43 (Figure 7B). Therefore, the PKC pathway might also be involved in the AGE-induced Cx43 upregulation in cardiomyocytes.

### 2.2. Discussion

Hyperglycemia is an independent risk factor and initiator for cardiac complications in diabetes, which has been reported to cause Cx43 down-regulation in various tissues, including endothelial cells and cardiomyocytes [7,22]. As a major gap junction protein, reduced Cx43 might affect GJIC function and cardiac conduction velocity, contributing to ventricular tachycardia and other arrhythmias. It would trigger mitochondrial shape change and cytochrome c release [23], finally leading to cell hypertrophy and apoptosis [24,25]. In our previous study, we also reported [6] that high glucose downregulated Cx43 protein level in neonatal rat cardiomyocytes. But, it still could not explain the discordant results of Cx43 expression in STZ-induced diabetic rat models [7–9]. There should be other mechanisms of elevating cardiac Cx43 expression in chronic diabetes. Recent evidence implicated that the accumulation of AGE in diabetes contribute to the pathogenesis of cardiovascular diseases [11,26]. Its effect on cardiac Cx43 expression, however, remains unknown.

In the present study, we found that Cx43 protein redistributed in AGE-infused rat heart and AGE significantly elevated Cx43 expression both *in vivo* and in cultured rat cardiomyocytes. The activation of PKC and Erk MAPK pathway might be involved in the mechanism. But, unexpectedly, the increased Cx43 did not enhance GJIC function.

Cx43 is the major gap junction protein in cardiomyocytes that mediates the function of cell coupling and leads to orderly electrical conduction [3]. The quantity alteration or redistribution of Cx43 contributes to various arrhythmias in diabetes. Its expression could be regulated at the transcriptional or post-transcriptional levels. Recently, Lin *et al.* [7] reported that in a three-week STZ-induced diabetic rat model, the total Cx43 amount and the immunoreactive particles for Cx43 at the intercalated disks were significantly decreased, but it could be alleviated by either lysosomal (NH4CL, Leupeptin) or proteasomal inhibitor (ALLN), which suggested that diabetes might cause proteolytic degradation of Cx43. It was consistent with another study [9] demonstrating the reduced Cx43 in a diabetic heart. Howarth *et al.* [8], however, found that in a 12-week diabetic rat model, total and phosphorylated Cx43 were elevated in ventricular myocytes, leading to prolongation of QRS and QT intervals. Therefore, the results of cardiac Cx43 remodeling still differed, which might be due to the miscellaneous factors in diabetic rat models. In diabetes, high glucose is regarded as a major initiator for cardiac complications. But, it also accelerates the formation and accumulation of AGE, which is the product of nonenzymatic glycoxidation of proteins and participates in various cardiovascular diseases. As high glucose has been previously recognized for its downregulation effect on cardiac Cx43 [6], the different results of Cx43 regulation might be partly due to the accumulation of AGE with the development of diabetes, which increased total Cx43 expression as our present study noticed.

We also demonstrated that AGE elevated both protein and mRNA levels of Cx43, indicating the transcriptional regulation, which differed from the possible post-transcriptional regulation of high glucose for not changing Cx43 mRNA level [6]. Our results suggested that besides high glucose, AGE might play an important role in cardiac electrical remodeling of diabetes. But, it was not consistent with the results of AGE on Cx43 in other cell lines. It has been demonstrated that in human hepatoma cell line (SKHep1) [27] and human aortic endothelial cells (HAEC) [28], the Cx43 protein was reduced by AGE treatment, while Rubenstein *et al.* [29] recently reported that AGE did not alter the Cx43 expression in endothelial cells. The different effect of AGE might be partly associated with the treated cell lines; even the proliferation rate differed in AGE-treated SKHep1 and HAEC.

We also noticed that Cx43 protein scattered and lost orderly distribution between intercalary discs in AGE-infused rat hearts. Recent studies [16,17] have already reported that disorganized connexins were associated with non-functional gap junctions and reduced cell excitability. And the increased lateral Cx43 was unlikely to enhance intercellular coupling, partly due to the possible dissociation of other junction proteins, such as *N*-cadherin and β-catenin. Therefore, the Cx43 upregulation by AGE might further contribute to conduction disturbance in a diabetic heart.

In our study, AGE did not affect the cell viability in neonatal rat cardiomyocytes determined by MTT assay, which was not consistent with the results in other cell lines. Sakata *et al.* reported that the low dosage of AGE did not produce any change in proliferation of smooth muscle cells (SMCs) at concentrations of up to 20 mg/L, but decreased that of SMCs at 40 mg/L [30]. In endothelial progenitor cells, co-culturing with AGEs (50–200 mg/L) also inhibited cell proliferation [31]. Another study, however, noticed that AGE accelerated cell proliferation assessed by MTT assay at 200 mg/L in cardiac fibroblast [32]. Therefore, the unremarkable change in cell viability in our present work might be partly due to the different cell type. But, AGE increased RAGE, the specific receptor for AGE, in a dose- and time-dependent manner and peaked at 200 mg/L for 24 h. In a 16-week diabetic rat model [33], the atria showed abundant expression of RAGE with diffuse interstitial fibrosis, which could be prevented by administration of an AGE formation inhibitor, OPB-9195. It was consistent with our results in AGE-infused rat heart, indicating RAGE protein upregulation by AGE treatment. But, in AGE-treated cardiomyocytes, RAGE did not show essential change during the first 12 h. In a recent report conducted by Sun *et al.*, they also demonstrated the time- and dose-dependent manner of RAGE expression by AGE treatment in endothelial progenitor cells, while it was only slightly elevated after 12 h [20]. In another study [34] about the effect of H_2_O_2_ on pancreatic cells, RAGE expression did not change after 6 h of H_2_O_2_ treatment, but it increased significantly after 24 h. To date, however, few studies mentioned the mechanism of RAGE elevation. It has been reported [35] that the RAGE promoter region had three potential NF-kappaB-like and two SP1 binding sites, indicating that RAGE activation might be associated with activation of the NF-kappaB pathway. Inactivated NF-kappaB is a latent form in the cytoplasm, which masks nuclear localization signals. Recently, Zhang *et al.* demonstrated that exposure of rat neonatal cardiomyocytes to Glycated-BSA (400 mg/L) increased nuclear NF-kappaB expression consistently after 24 h, which suggested the enhanced translocation of NF-kappaB to nucleus [19]. Therefore, it could be a limitation for the lack of investigation for NF-kappaB expression and its activating time in our study. It might be possible that RAGE acted more as a link to AGE and engaged downstream signal transduction pathways, which in turn upregulated RAGE expression and amplified these cellular effects. Recently, many studies [12–14] focused on the pathogenic effect of AGE-RAGE system in cardiomyocytes. We used siRNA technology to knock down RAGE expression, and it abrogated the upregulation effect of AGE on Cx43. Therefore, AGE might show its effect on cardiac Cx43 by RAGE activation.

Unexpectedly, the cellular gap junction function was not enhanced by AGE treatment. It was believed that function of cardiac gap junction depended on multiple levels involving Cx43 expression, phosphorylation and degradation [36,37]. But, in recent studies, the effect of Cx43 phosphorylation on cell coupling still differed in various cell types [38,39]. Some findings demonstrated that phosphorylated Cx43 acted as the main functional form of Cx43 and dephosphorylation could impair the electrical coupling and signal transmission through gap junction [40–42]. In our study, AGE increased both non-phosphorylation and phosphorylation expression of Cx43, and the possible mechanism was the activation of the PKC and Erk MAPK pathways. Erk was reported to be critical for cardiac Cx43 upregulation and increased GJIC [43]. However, PKC-induced Cx43 Ser368 phosphorylation resulted in disassembly of the gap junction and reduced channel conductance [39,44], indicating the negative effect of GJIC by PKC activation. It might partly explain the unchanged GJIC function by AGE treatment in spite of Cx43 upregulation. It was also believed that the phosphorylation sites of Cx43 could affect their justified location and membrane association. A recent study [7] further pointed out that Cx43 was redistributed into cell periphery or cytoplasm in diabetes, leading to the suppression of intercellular impulse propagation. Therefore, the AGE-increased Cx43 proteins were possibly unable to assemble properly to exert their activity, which contributed to the unremarkable change of GJIC function.

The present study demonstrated that AGE-RAGE system elevated Cx43 expression both *in vitro* and *in vivo*. Further work will still be required to determine the phosphorylation sites of Cx43 and their corresponding effect in a diabetic heart.

## 3. Experimental Section

### 3.1. Chemicals and Reagents

AGE was prepared by incubating bovine serum albumin (BSA, 50 mg/mL) with D-glucose (0.5 M) in phosphate buffered saline (PBS, 0.2 M, pH 7.4) for 12 weeks at 37 °C. Unincorporated glucose was dialyzed extensively overnight against PBS. Unmodified BSA was prepared under the same conditions without glucose as a control. As other report described [45], AGE was identified by a fluorescence spectrophotometer (Hitachi, Tokyo, Japan) using a 440 nm emission wavelength on excitation at 370 nm, which confirmed the higher intensity of AGE in AGE-modified BSA than that in unmodified BSA. The AGE solution was filtered to be sterile by 0.22 μM Millex GP filter unit (Millipore, Billerica, MA, USA) and confirmed to be endotoxin free (<2.5 μL of endotoxin) by using an endotoxin testing kit (Chromogenic TAL Endpoint Assay Kit, Xiamen, China). Receptor for AGE (RAGE) polyclonal antibody was obtained from Chemicon (Temecula, CA, USA). Monoclonal rabbit antibodies, including anti-Erk, anti-phospho-Erk, anti-JNK, anti-phospho-JNK, anti-p38, anti-phospho-p38 and anti-phospho-PKC α/β2, were purchased from Cell Signaling Technology (Boston, MA, USA). PKC polyclonal antibody was obtained from Santa Cruz Biotechnology (Santa Cruz, CA, USA). The corresponding selective inhibitors, including PD98059, SP600125, SB203580 and GF109203X, were obtained from Merck (Darmstadt, Germany). 3-(4,5-dimethylthiazol-2-yl)-2,5-diphenyl-tetrazolium bromide (MTT), Lucifer yellow dye and Cx43 polyclonal antibody were obtained from Sigma (St. Louis, MO, USA). HRP-marked anti-glyceraldehyde-3-phosphate dehydrogenase (GAPDH) antibody was supplied by Kangchen (Shanghai, China).

### 3.2. AGE-Infused SD Rat Model

Male SD rats aged eight weeks and weighed 200 g were obtained from the experimental animal center of Zhejiang University. Rats were injected intraperitoneally (i.p.) with BSA alone or AGE-BSA at a dosage of 40 mg/kg/d for 28 days (*n* = 5 per group) [46,47]. At the end of the experiments, rats were sacrificed, and hearts were collected for immunohistochemisty. Animal procedures were performed according to the *Guide for the Care and Use of Laboratory Animals* (NIH, revised 1996), and approval was granted by the university ethics review board.

### 3.3. Immuohistochemisty

Hearts were fixed and embedded in paraffin. Samples were then cut into 4-μM sections and used for immuohistochemisty study, including RAGE (1:500) and Cx43 (1:2000) staining, respectively. They were followed by incubation with specific HRP-marked second antibodies and DAB (Dojindo, Kumamoto, Japan). After PBS rinsing, samples were counterstained in hematoxylin, dehydrated and mounted. The Cx43 and RAGE expressions were analyzed by ImageJ 1.43u software (http://rsb.info.nih.gov/ij) (National institutes of Health, Bethesda, MD, USA).

### 3.4. Cell Culture

Neonatal rat cardiomyocytes were cultured, as we previously described [6]. Briefly, the heart tissue isolated from the heart of 1–2 day old Sprague-Dawley rats (provided by the experimental animal center of Zhejiang University) were dissected and digested in a Ca^2+^ and Mg^2+^ free PBS containing 0.1% trypsin and 0.1% type II collagenase for 10 min at 37 °C. DMEM cell culture medium (glucose concentration: 5.5 mM) containing 10% fetal bovine serum (FBS) was added into the collected cell supernatant to stop the digestive effect of trypsin and collagenase. The above steps were repeated until the tissues were completely digested. The cell suspension was then centrifuged and suspended in DMEM cell culture medium with 10% FBS in a humidified 5% CO_2_/95% air atmosphere at 37 °C. The fibroblast in the cell suspension was reduced by pre-plating for 1 h due to the differential cell adhesion [48] and the addition of 5′-BrdU (0.01mM) during the first three days to inhibit the growth of fibroblast.

### 3.5. Cell Viability Assay

Cells were seeded at 2 × 10^4^ cells/well in 96-well culture plates and incubated in serum-free medium for 24 h before experiment. The cardiomyocytes were then treated with AGE at various concentrations for 24 h or by various incubation periods. The MTT solution (final concentration, 0.5 mg/mL) was added to each well for an additional 4 h at 37 °C. The supernatants were then removed, followed by 200 μL dimethyl sulfoxide (DMSO) added into each well. The absorbance was read at a wavelength of 490 nm. Each independent experiment was repeated five times.

### 3.6. Western Blot Assay

Cells in 6-well plates were harvested and washed with PBS for three times, followed by lysing on ice in 100 μL RIPA solution containing 50 mM Tris (pH 7.4), 150mM NaCl, 1% NP-40, 5% deoxycholic acid, 0.1% SDS, 1 mM EDTA, 10 mM NaF, 1 mM Na_3_VO_4_, 1 mM dithiothreitol, 1 mM PMSF, 2 μg/mL leupeptin for 30 min. Proteins were quantified with the Bio-Rad DC Protein Assay Kit II (Bio-Rad, Hercules, CA, USA). Samples containing equal amounts of protein were run on a 10% tris-glycine gradient gel, transferred to PVDF membranes and blocked for 1 h with 5% nonfat milk in TBST (Tris-buffered solution containing 0.1% Tween 20) at room temperature. Membranes were then soaked with primary antibodies overnight at 4 °C followed by secondary antibody incubation for 1 h at room temperature. Finally, the membranes were reacted with enhanced chemiluminescence (ECL) reagents and exposed by Image Quant LAS-4000 (Fujifilm, Tokyo, Japan). Band densities were determined by an image Multi-Gauge Software (Fujifilm, Tokyo, Japan). Each experiment was repeated at least 3 times.

### 3.7. Real-Time Reverse Transcription-PCR (Real-Time RT-PCR)

TRIZOL reagent (Invitrogen, Carlsbad, CA, USA) was used to isolate total RNA, which was converted into cDNA by murine leukemia virus reverse transcriptase (Promega, Mannheim, Germany). The primers for amplification of specific rat mRNA were as follows: Cx43 (116 bp), 5′-TCTGCCTTTCGCTGTAACACT-3′ and 5′-GGGCACAGACACGAATATGAT-3′; RAGE (139 bp), 5′-CAGGGTCACAGAAACCGG-3′ and 5′-ATTCAGCTCTGCACGTTCCT-3′; GAPDH (152 bp), 5′-ATTCTCCCACGGCAAGTT-3′ and 5′-CGCCAGTAGACTCCACGACATA-3′ (sense and antisense, respectively). Real-time PCR was performed on the ABI 7500 cycler (Applied Biosystems, CA, USA) using SYBR Premix Ex Taq reagent (Takara, Shiga, Japan). The relative amount of target gene expression was normalized by GAPDH as an internal control.

### 3.8. Scrape Loading Dye Transfer Assay

The function of gap junctional intercellular communication (GJIC) was assessed by scrape loading dye transfer assay as previously described. Cells cultured in 35 mm dishes were incubated in preheated (37 °C ) 1% Lucifer Yellow dye solution (dissolved in Ca^2+^ and Mg^2+^ free PBS) after they were rinsed with warm PBS (37 °C ). A surgical blade was used to make scrape lines through the cell monolayer. After 3 min, the dye solution was removed. The cells were rinsed with PBS and fixed by 4% paraformaldehyde for 1 min. The distance of the dye transferred from the scraped margin was observed with an inverted fluorescent microscope.

### 3.9. Small Interference RNA Transfection

Small interfering RNA molecules (siRNA) were designed by GenePharma (Shanghai, China). The siRNA (Up: 5′-GCUAGAAUGGAAACUGAACTT-3′; Down: 5′-GUUCAGUUUCCAUUCUAGCTT-3′) targeted at RAGE mRNA were used to knock down the expression of RAGE. A scrambled siRNA (Up: 5′-UUCUCCGAACGUGUCACGUTT-3′; Down: 5′-ACGUGACACGUUCGGAGAATT-3′) was designed as a negative control. The siRNA diluted in Hiperfect transfection reagent (Qiagen, Hilden, Germany) was premixed with serum-and antibiotic-free DMEM medium at room temperature for 10 min. Then, the cells in 6-well plates were incubated with 500 μL mixed solution containing 100 nM siRNA, followed by additional 500 μL culture medium after 4 h. 24 h later, the culture medium was replaced by DMEM medium with penicillin/streptomycin and 10% FBS. Experiments were carried out 48 h after completion of the transfection.

### 3.10. Statistical Analysis

Results were expressed as mean ± SEM. for at least three individual experiments. Statistical analysis between groups was performed by one-way ANOVA. The level of *p* < 0.05 was considered statistically significant.

## 4. Conclusions

In conclusion, the present study demonstrated that AGE-RAGE system elevated Cx43 expression both *in vitro* and *in vivo*. The phosphorylation of the PKC and Erk MAPK pathways were involved in this process. The increased Cx43 expression did not enhance cellular coupling, and they also lost orderly distribution along intercalary discs *in vivo* models, which might contribute to electrical remodeling in a diabetic heart. Further work will still be required to investigate the phosphorylation sites of Cx43 and the possible Cx43 redistribution in cultured cardiomyocytes.
